# AI-Powered Thermal Fingerprinting: Predicting PLA Tensile Strength Through Schlieren Imaging

**DOI:** 10.3390/polym18030307

**Published:** 2026-01-23

**Authors:** Mason Corey, Kyle Weber, Babak Eslami

**Affiliations:** Mechanical Engineering Department, Widener University, Chester, PA 19013, USAkeweber@widener.edu (K.W.)

**Keywords:** 3D printing, schlieren imaging, machine learning, FDM, material characterization

## Abstract

Fused deposition modeling (FDM) suffers from unpredictable mechanical properties in nominally identical prints. Current quality assurance relies on destructive testing or expensive post-process inspection, while existing machine learning approaches focus primarily on printing parameters rather than real-time thermal environments. The objective of this proof-of-concept study is to develop a low-cost, non-destructive framework for predicting tensile strength during FDM printing by directly measuring convective thermal gradients surrounding the print. To accomplish this, we introduce thermal fingerprinting: a novel non-destructive technique that combines Background-Oriented Schlieren (BOS) imaging with machine learning to predict tensile strength during printing. We captured thermal gradient fields surrounding PLA specimens (*n* = 30) under six controlled cooling conditions using consumer-grade equipment (Nikon D750 camera, household hairdryers) to demonstrate low-cost implementation feasibility. BOS imaging was performed at nine critical layers during printing, generating thermal gradient data that was processed into features for analysis. Our initial dual-model ensemble system successfully classified cooling conditions (100%) and showed promising correlations with tensile strength (initial 80/20 train–test validation: R^2^ = 0.808, MAE = 0.279 MPa). However, more rigorous cross-validation revealed the need for larger datasets to achieve robust generalization (five-fold cross-validation R^2^ = 0.301, MAE = 0.509 MPa), highlighting typical challenges in small-sample machine learning applications. This work represents the first successful application of Schlieren imaging to polymer additive manufacturing and establishes a methodological framework for real-time quality prediction. The demonstrated framework is directly applicable to real-time, non-contact quality assurance in FDM systems, enabling on-the-fly identification of mechanically unreliable prints in laboratory, industrial, and distributed manufacturing environments without interrupting production.

## 1. Introduction

Fused deposition modeling (FDM) is among the most widely used techniques in additive manufacturing. This is due to its cost effectiveness and versatility within the industry. The process involves heating plastic filament to a semi-molten state and then depositing it layer by layer. As layers accumulate, a three-dimensional object is created directly from a digital model. This method allows for the creation of complex geometries, such as lattices, that would be difficult, if not impossible, to replicate with traditional techniques. In addition to enabling the fabrication of complex geometries, FDM offers a range of other technical advantages, such as its widespread compatibility with a large range of materials. Compatibility with inexpensive and biodegradable materials like PLA to engineering-grade polymers such as ABS, PETG, and PEEK allows the process to be tailored to diverse application requirements. Another technical advantage of FDM over traditional manufacturing techniques is its low material waste compared to subtractive manufacturing processes. This ultimately reduces production costs and supports sustainable manufacturing practices. However, despite its many advantages, FDM lacks a cost-effective, non-contact method for capturing the convective thermal environment that governs interlayer bonding and mechanical reliability.

Recent studies emphasize that FDM’s layer-wise deposition inherently produces anisotropic microstructures, where mechanical performance depends strongly on thermal history during solidification and cooling [1]. The temperature distribution in the extruded bead controls inter- and intra-layer bonding through polymer chain mobility and reputation, meaning that even slight variations in cooling rate can cause substantial differences in bonding quality [2]. As a result, the thermal environment surrounding the print influences porosity formation, residual stresses, and ultimately failure modes—factors often overlooked in parameter-only models.

Overall, the technical advantages of FDM have allowed it to have a profound impact on industrial manufacturing. One of its most significant contributions is expanding the use of rapid prototyping beyond only specialized facilities. Automotive, aerospace, and consumer electronic industries have adopted FDM to significantly reduce product development costs and timelines, which enables engineers to move from a digital concept to a physical prototype in very short periods of time. A result of this accelerated process is lower development costs and increased number of design alternatives that can be tested before final production. In 2024, prototyping accounted for roughly 48.3% of global FDM printer sales, proving the technology’s key role in accelerating product development. However, market analyses show that the production of end-use parts is the fastest-growing application segment, with a projected compound annual growth rate of 23.7% from 2025 to 2030 [3]. This shift represents a broader industrial trend of moving FDM beyond prototyping into production applications.

Consequently, the technical advantages and broad industrial impacts of FDM have allowed it to emerge as the most widely adopted technique in additive manufacturing. A 2024 industry survey revealed that FDM is the most frequently used additive manufacturing technology, with 59% of respondents reporting it as their primary method [4]. Additionally, as a result of its widespread adoption, the global market for FDM was valued at approximately USD 1.7 billion in 2023 and is projected to reach nearly USD 6.9 billion by 2030, reflecting a well above average compound annual growth rate of 21.8% [4].

Despite this widespread adoption and clear advantages, FDM still faces significant challenges, particularly in the variability in mechanical properties and the absence of in situ quality control methods. Mechanical properties of FDM parts are highly variable due to dependence on interlayer bonding and the fact that environmental conditions during printing, such as ambient temperature, airflow, and humidity, can significantly influence these mechanical properties. Even when printing parameters are nominally identical, studies have reported strength variations exceeding 10% between parts, highlighting the sensitivity of FDM to subtle changes in environmental conditions [5]. This variability poses challenges for using FDM in applications that demand consistent and predictable performance.

Addressing these challenges requires methods that can capture the thermal environment during printing without destructive testing. Our research introduces the use of Schlieren imaging to analyze environmental thermal patterns to find “thermal fingerprints” that ultimately predict tensile strength. Schlieren imaging is a technique that analyzes tiny refractions of light within the air to reveal a density gradient. Specifically, we used Background-Oriented Schlieren (BOS) imaging. For BOS imaging, a reference photo is taken of a background of randomly generated dots. Then, a phase object (anything that changes the density of the air, such as heat, pressure, velocity, etc.) is introduced. When the test photo is taken, light will refract differently through the varying densities of air. This will cause some dots to “shift” within the test image. These shifts can be reverse-engineered to construct a density gradient.

Thermal fluctuations are one of the most significant contributors to mechanical inconsistency in FDM parts. Researchers have shown that polymer cooling profiles directly influence interlayer diffusion depth and the degree of molecular entanglement, which determines final tensile strength [6]. Additionally, ambient airflow disturbances have been experimentally linked to variations in bead geometry, void formation, and localized hotspots, each degrading the uniformity of mechanical performance. These findings reinforce the need for real-time thermal monitoring to capture print-specific thermal histories.

Infrared thermography (IR) is an existing quality assurance (QA) technique that enables non-contact, real-time thermal mapping and has been found to be effective for detecting anomalies and defects in FDM [7]. The accuracy of these systems depends largely on calibration, camera frame rate/resolution, and line-of-sight [8]. Unfortunately, conventional IR primarily measures surface temperature. It does not directly capture thermal gradients in the surrounding air that influence cooling, and systems with sufficient speed/resolution can be costly. Therefore, while IR thermography remains one of the most widely investigated approaches for monitoring thermal conditions in FDM, its reliance on surface-emitted radiation fundamentally limits its ability to detect convective effects in the surrounding air that govern cooling [9]. Moreover, emissivity variation between materials, colors, and surface finishes introduces additional calibration challenges, reducing accuracy for low-cost or consumer-grade systems [10]. These limitations highlight the need for optical refraction-based approaches capable of visualizing air-side thermal gradients.

Ultrasonic testing (UT) is another existing QA technique and can reveal internal defects (voids, lack-of-fusion, poor bonds) and has been applied to AM polymers and composites. Reviews conclude UT is effective for subsurface inspection, depending on access and geometry. However, conventional contact UT requires the tool to be touching the specimen. As a result, complex geometries can complicate signal interpretation. Laser UT reduces coupling issues but increases system complexity [11]. Therefore, routine use is very challenging due to coupling, access, fixture constraints, and high implementation cost.

X-ray computed tomography (XCT) provides high-fidelity, volumetric inspection of internal structure (porosity, lack-of-fusion, dimensional accuracy) and is widely used for qualification and research across AM. Reviews state that XCT is precise but also slow and resource-intensive. Additionally, scan/reconstruction times and uncertainty are both negatives, especially for larger parts or lattice structures. Therefore, this process is largely implemented post-process rather than in situ [12]. It is also constrained heavily by high operating costs. Other in situ sensing technologies such as acoustic emissions and optical coherence tomography (OCT) have also been explored, yet they face similar constraints. Acoustic methods are highly sensitive to machine vibrations and require sophisticated filtering to isolate defect-relevant signals [13]. OCT offers micron-scale subsurface imaging but its penetration depth in polymers is limited, and real-time integration remains cost-prohibitive. Consequently, there is still no cost-effective, fully non-contact technique capable of measuring convective thermal behavior during FDM.

Optical and flow-based diagnostics offer a method of observing the surrounding thermal environment, or “thermal fingerprint,” of a print that determines cooling, interlayer bonding, and ultimately mechanical properties in FDM. Classic Schlieren methods are well-established for visualizing refractive-index gradients related to temperature changes, and they provide the conceptual foundation for modern Background-Oriented Schlieren (BOS) approaches. BOS has gained significant attention because it can quantify refractive-index gradients corresponding to temperature fields with minimal hardware cost. Unlike classical Z-type Schlieren systems, BOS eliminates the need for mirrors and knife edges, making it particularly suited for compact or cluttered environments such as FDM printer enclosures [14]. Recent research shows that BOS sensitivity can approach that of traditional Schlieren when using high-frequency dot patterns and sub-pixel correlation algorithms [15]. These advances enable BOS to capture subtle convective structures that conventional thermographic approaches overlook. Recent reviews demonstrate how BOS has developed into a versatile tool, including tomographic reconstructions, event-based imaging, and improved algorithms. BOS has expanded its reach from purely aerodynamics to heat-transfer and manufacturing settings [13].

BOS performance depends on background pattern design and displacement-extraction algorithms [16]. Recently, physics-informed learning has been combined with Tomo-BOS to infer 3-D velocity and pressure fields from temperature snapshots, which validates BOS-based data collection against Particle Image Velocimetry (PIV) [17]. Importantly, for additive manufacturing, optical Schlieren systems have already been adapted to laser-based processes, where they capture thermal-flow signatures during deposition. This is evidence that refractive-index physics translates to manufacturing environments [18]. Together, these works justify BOS-based “thermal fingerprinting” as physically feasible and practically deployable for real-time quality assurance in polymer additive manufacturing.

The application of machine learning (ML) to manufacturing has long been recognized as a means to address the complexity, variability, and uncertainty in production systems [19]. In regards to additive manufacturing, several publications have investigated machine learning models that use print process parameters such as temperature, speed, layer thickness, and infill as inputs to predict quality or optimize printing [20,21,22].

For example, ML models are developed to correlate laser powder bed fusion process parameters to mechanical properties of printed parts, demonstrating that parameter variations alone can be predictive of quality outcomes (without explicitly modeling thermal fields) [20]. Similarly, Qian et al. (2025) apply ML-assisted optimization of binder jetting printing parameters for aluminum alloys, showing that tuning parameters via learned models improves part quality [21]. Wang et al. (2024) use an LSTM neural network to predict mechanical properties from 3D printing parameter settings [22].

Regarding FDM specifically, recent work has shown that ML models can predict mechanical performance from FDM process parameters. Nikzad et al. trained and compared multiple algorithms using inputs such as nozzle temperature, layer height, speed, and infill to predict ultimate tensile strength (UTS) of PLA parts [23]. They reported strong predictive accuracy and highlighted that parameter-only models can provide actionable guidance without in situ sensors. Similarly, Charalampous et al. presented ML models that predict tensile properties of FDM parts from input parameters and material selections, demonstrating good fit across algorithms and offering model interpretation for parameter influence [24].

In addition to parameter-based prediction models, several studies have explored integrating sensor data with machine learning to improve prediction accuracy. Thermal-camera-driven deep learning has been shown to detect defects such as under-extrusion and delamination in real time [9]. Similarly, vibration and acoustic emissions have been fused with ML models to predict extrusion anomalies, but these signals are highly machine-dependent and require complex preprocessing. Despite these efforts, no prior work has integrated BOS-derived volumetric thermal data with ML, leaving a major research gap in physically grounded, air-side thermal monitoring for FDM.

Together, these parameter-centric ML studies show that nominal print settings such as layer height, speed, temperature, and infill can serve as a practical baseline for predicting and optimizing FDM part properties. However, these parameter-based ML models implicitly assume that identical process settings produce equivalent thermal histories, an assumption violated by environmental airflow, enclosure effects, and machine-specific convection. As a result, these models cannot capture part-specific thermal histories that directly govern interlayer diffusion and bond formation. By directly modeling those fields via Schlieren-based thermal fingerprints, our work moves beyond parameter tables to a more physically grounded path toward real-time quality assurance and property prediction.

Prior research underscores that the absence of in situ thermal data is one of the primary barriers preventing FDM from achieving industrial-grade reliability [25]. Predictive models that rely solely on parameter settings cannot account for environmental disturbances, machine-specific airflow patterns, or part-specific convective behaviors. Therefore, developing a sensing framework that captures the true thermal environment—not merely the commanded process parameters—remains a major open challenge. The primary objective of this research is to develop a non-destructive, non-invasive system capable of predicting the tensile strength of PLA parts during FDM printing by analyzing thermal gradients. To accomplish this, this study relies on the concept of thermal fingerprinting. Thermal fingerprinting is the idea that the thermal gradients of the air around an FDM print (driven by localized heating and cooling) contain a unique signature or “fingerprint.” These patterns can then be analyzed to reveal valuable information about the resulting mechanical properties of a print. By leveraging this concept, this study aims to demonstrate how this real-time thermal data can be transformed into a reliable indicator of the performance of prints.

In this context, thermal fingerprinting refers to the identification of unique, part-specific thermal gradient patterns that emerge during deposition and cooling and that encode information about interlayer bonding quality. Unlike parameter-based descriptors, these fingerprints arise from the actual convective and thermal environment experienced by each print. When combined with machine learning, these thermally derived signatures enable data-driven inference of mechanical properties directly from in situ measurements rather than from nominal process settings. This coupling of Schlieren-based thermal field visualization with artificial intelligence motivates the title of this work: AI-powered thermal fingerprinting for tensile strength prediction in FDM-printed PLA.

In order to achieve this, Background-Oriented Schlieren (BOS) imaging was explored as a low-cost and non-intrusive technique for capturing thermal fingerprints during FDM. Unlike conventional non-destructive testing techniques such as ultrasound, X-ray CT, or IR thermography, BOS imaging requires only a simple optical setup and can be integrated into the printing process itself. This allows for the continuous capture of thermal gradients at critical layers, offering a real-time perspective on part quality without interrupting production. The ability to monitor thermal history in situ provides a significant step toward closing the gap in quality assurance for FDM.

A key contribution of this work is demonstrating that the entire experimental setup minus the camera can be constructed for under USD 90, using readily available consumer components: two standard hairdryers (~USD 20 each), acrylic mounting sheets (~USD 10), and a single softbox LED light (~USD 40). This ultra-low-cost configuration demonstrates the accessibility and scalability of the system, unlike infrared or X-ray setups that require specialized hardware costing thousands of dollars. By relying on inexpensive and modular components, this framework enables universities, small research labs, and even hobbyist makerspaces to replicate thermal fingerprinting experiments without prohibitive financial barriers. The low entry cost directly supports broader adoption of real-time quality monitoring in FDM, aligning with the study’s objective to democratize advanced manufacturing research tools.

This study introduces the first application of Background-Oriented Schlieren (BOS) imaging for quality assessment in polymer additive manufacturing. Unlike prior parameter-based or surface-temperature monitoring approaches, this work captures volumetric, air-side thermal gradients surrounding the print and integrates them with machine learning to predict tensile strength. The proposed concept of thermal fingerprinting establishes a physically grounded, non-contact, and low-cost pathway for in situ quality prediction that directly reflects the true thermal environment experienced during FDM printing. The specific objectives of this work are to 1. Develop a low-cost BOS imaging setup capable of capturing convective thermal gradients during FDM printing of PLA. 2. Quantify thermal fingerprint features that describe spatial, directional, and temporal characteristics of the air-side thermal environment. 3. Evaluate whether Schlieren-derived thermal features can reliably classify environmental printing conditions. 4. Assess the feasibility of predicting ultimate tensile strength of PLA specimens using machine learning models trained on thermal fingerprints. 5. Identify limitations related to dataset size and generalization to inform future scaling of in situ quality assurance frameworks.

## 2. Materials and Methods

Printing System and Parameters: All test specimens were created using a Prusa i3 K3S+ printer (Prusa Research, Prague 7, Czech Republic) with PLA filament of 1.75 mm diameter manufactured by Overture with the required nozzle temperature of 190–220 °C with the reported tensile strength around 47 MPa in X and Y directions. Printing parameters were constant throughout printing to ensure consistency. They included a nozzle temperature of 200 °C, heated bed temperature of 60 °C, layer height of 0.2 mm, print speed of 60 mm/s, and infill density of 20%. These parameters were selected due to their prevalence in consumer-grade additive manufacturing applications.

**Figure 1:**
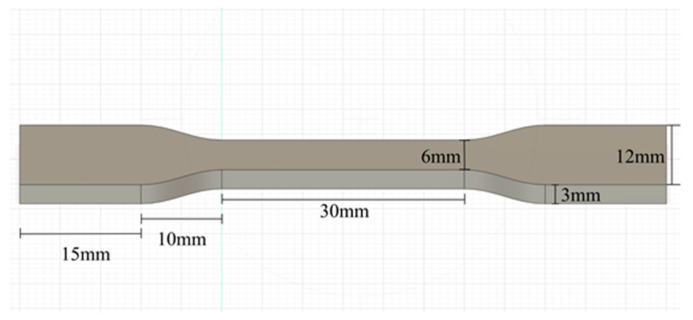
Dogbone CAD Model: ASTM D638 [26] tensile test sample with dimensions.

“Dogbone” test specimens were designed with overall dimensions of 80 mm in length. The gauge section measures 30 mm × 6 mm × 3 mm (length × width × thickness). All specimens were printed in a vertical orientation (build direction parallel to specimen length) to maximize the imaging field of view during testing. This orientation resulted in tensile loading applied parallel to the printed layers. Given the 0.2 mm layer height, each 80 mm specimen comprised 400 layers, as shown in Figure 1.

Environmental Conditions: six distinct environmental conditions were designed to evaluate the effects of thermal gradients and airflow patterns on specimen behavior during printing. The conditions are explained in Table 1.

For each environmental condition, five specimens were printed to ensure some statistical validity and reproducibility (*n* = 5 per condition, total *n* = 30). Specimens were labeled by combining the condition letter with a number (A1–A5, B1–B5, …, F1–F5). This system allowed for data organization and integration with machine learning algorithms for analysis.

Schlieren Imaging System: A Background-Oriented Schlieren (BOS) imaging system was implemented to visualize thermal gradients and airflow patterns during specimen printing. The system was adapted from standard BOS configurations to accommodate space constraints while maintaining measurement accuracy. The imaging system had four primary components: a patterned background target, LED illumination, the test subject (3D printer), and a digital camera positioned 2 m away.

The background pattern consisted of randomly distributed dots generated using a custom Python algorithm (Python 3.14.2), with final dimensions of 2000 × 1500 pixels printed on A1 format paper. The dot pattern used a density coefficient of 0.2 (normalized scale 0–1) to optimize tracking resolution while preventing pattern saturation.

**Figure 2:**
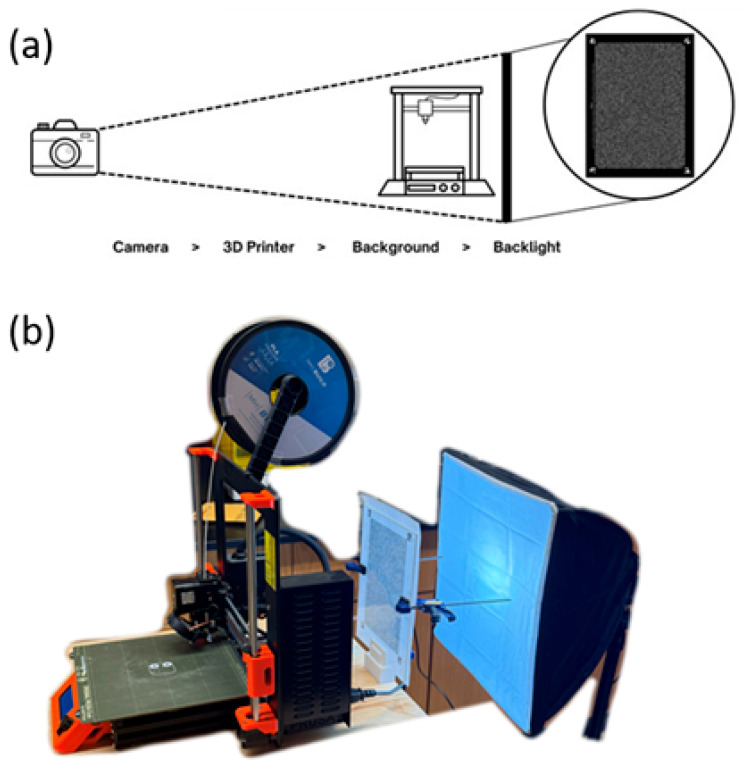
(**a**) Schlieren imaging experimental setup diagram and (**b**) real experimental setup.

A key modification from conventional Schlieren systems involved the implementation of an inverted contrast scheme: white dots on a black background rather than the traditional black-on-white configuration. This inversion enhanced digital masking capabilities during image processing operations, that will be discussed in future sections of this work (Figure 2).

Uniform backlighting was achieved using an LED light positioned behind the target background. The 3D printer was positioned between the illuminated background and a Nikon D750 digital camera stationed 2 m from the test field. The camera optical system was focused on the background plane to maximize dot pattern sharpness, while the LED backlighting provided sufficient contrast to enable high-resolution dot displacement tracking.

Images were captured at nine predetermined layer heights that correspond to critical strength transitions in the specimen fabrication process:

- Layers 125, 135, 145: Transition zone entry (beginning of testing section);
- Layers 175, 200, 225: Uniform gauge section region;
- Layers 250, 265, 275: Transition zone exit (end of testing section).

These specific layers were selected to capture thermal field evolution during critical estimated breaking points. The protocol yielded 9 images per specimen across all 6 environmental conditions (A–F) and 5 specimens per condition, resulting in a total dataset of 270 high-resolution thermal field images for analysis.

Data Processing and Feature Engineering: The BOS image processing workflow was designed to address the challenges of extracting meaningful thermal gradient information from Schlieren images containing varying pattern distributions and signal-to-noise ratios. The approach used an adaptive pattern detection algorithm that automatically selected the optimal processing strategy based on analysis of background pattern characteristics.

The study employed an inverted contrast scheme (white dots on black background) rather than conventional black-on-white patterns for three critical reasons. First, white-on-black patterns enable superior digital masking during image processing because binary operations can directly isolate trackable regions using simple intensity thresholding (pixels > 200 on 0–255 scale). Second, the inverted contrast reduces the needed processing power by allowing immediate identification of analysis regions without complex morphological operations required for dark feature extraction. Third, LED backlighting provides enhanced contrast for white dots, improving signal-to-noise ratios for optical flow calculations compared to traditional illumination of darker features.

The processing algorithm created binary masks by detecting white regions present in both reference and test images, ensuring optical flow calculations were restricted to areas containing trackable patterns in both frames. This dual-image masking approach was essential because the structures of the 3D printer and specimen would disrupt the program and lead to analyzing mismatched regions that introduce inaccurate flow vectors unrelated to actual thermal gradients.

Feature Extraction: The feature extraction methodology was designed to capture multi-scale thermal characteristics while still having resistance to noisy thermal environments that are inevitable in additive manufacturing processes. The methodology addressed three challenges: (1) spatial diversity of thermal fields, (2) temporal evolution over time, and (3) position invariance. This enabled generalization across the printer configurations in our dataset.

**Figure 3:**
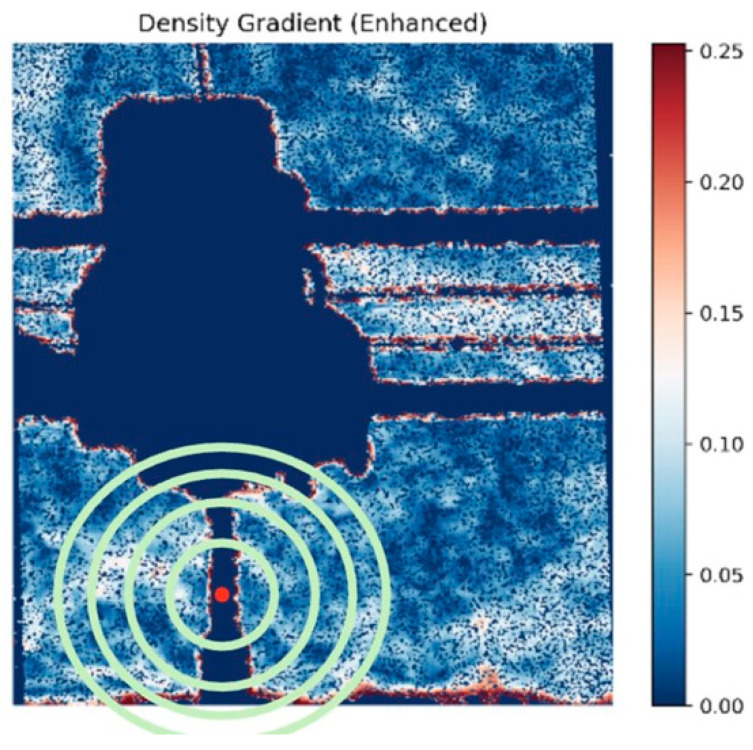
ML Radial Zones: defined location for the center of the zone with 20%, 40%, and >60% radial distances.

To ensure that feature comparison was consistent across all images, thermal fields were analyzed and compared to each other in radial zones centered on a user-assigned print center. Concentric zones at 20%, 40%, 60%, and >60% of maximum radial distance provided distance-normalized spatial sampling. This ensured that the sampling was independent of absolute image coordinates or subtle changes in print positioning (Figure 3).

Within each spatial zone, we were able to quantify thermal gradient distributions: central tendencies (mean, median), variability measures (standard deviation, interquartile range), distributional shape (skewness), and extreme values (95th percentile, maximum magnitude). These statistics captured different aspects of thermal behavior. For example, means indicated average thermal activity, standard deviations quantified thermal variability suggesting process stability, and skewness revealed asymmetric thermal distributions characteristic of specific cooling conditions.

Directional flow components (u, v vectors) received separate analysis because thermal gradients show strong correlations between cooling airflow patterns and direction. Asymmetry methods compared left-right and top-bottom thermal magnitudes, providing numerical measures of spatial thermal gradients that are critical for distinguishing asymmetric environmental conditions. The horizontal gradient index (left_mean−right_mean)/(left_mean+right_mean) provided asymmetry results that were resistant to overall thermal magnitude variations.

Temporal features tracked thermal evolution across the nine critical layer heights, capturing process dynamics unable to be seen with single-layer analysis. Layer selection targeted three geometric phases: gauge section entry (layers 125–145), uniform printing region (layers 175–225), and gauge section exit (layers 250–275). This sampling strategy ensured that thermal transients during geometric transitions were captured, as well as thermal patterns during uniform deposition.

More forms of feature extraction included quantifying how the thermal environment changed over time with linear trend fitting across layers and coefficient of variation calculations to look at thermal environment consistency over time. We validated this data with correlation analysis to identify redundant features. We utilized variance analysis to eliminate any near-constant features. Features showing excessive correlation (r > 0.95) were flagged for potential removal.

Mechanical testing followed standard tensile testing protocol with modifications appropriate for additively manufactured specimens and the specific research objectives. The testing methodology was designed to provide accurate tensile strength measurements while minimizing experimental variability that could overcomplicate finding correlations with thermal gradient features. Visual inspection identified specimens with visible layer delamination, surface defects, or printing artifacts. No samples in our dataset had visible above-mentioned defects. The vertical printing orientation (stress applied parallel to layer interfaces) made specimens particularly susceptible to interlayer bonding defects that could cause premature failure unrelated to bulk material properties.

Tensile testing utilized an ADMAT testing machine with a 1000 mm/min speed. Ultimate tensile strength was determined as the maximum stress sustained during testing, calculated from peak load divided by cross-sectional area. Quality control criteria included visual inspection of fracture surfaces to identify specimens failing due to layer delamination versus bulk material failure. No samples in our dataset had unrelated failures.

Machine Learning Classification Model: We used an ensemble of Random Forest and XGBoost classifiers to predict environmental conditions from thermal gradient patterns. Random Forest handled the 1847 initial feature space effectively with limited samples, while XGBoost’s gradient boosting corrected systematic errors through sequential learning. The ensemble combined predictions through majority voting to provide clear classification decisions.

To prevent overfitting with only 30 specimens, we reduced the feature space using SelectKBest with ANOVA F-statistics. We selected the 40 most prominent features for prediction, achieving a ratio of 0.75 specimens per feature. The selected features captured three key thermal characteristics: spatial asymmetry (horizontal gradients indicating left-right heating differences), temporal evolution (thermal buildup rates distinguishing heating from cooling), and radial profiles to organize data/patterns.

We split the data 80/20 for training and testing using stratified sampling to ensure all six cooling conditions were represented in both sets. This was critical because equal specimen counts per condition (*n* = 5) could otherwise exclude entire conditions from training or testing (Table 2).

We also assessed model performance using 5-fold cross-validation with stratified sampling. Each fold was trained on 24 specimens and validated on 6. We evaluated models using overall accuracy and confusion matrices to identify misclassification patterns.

The regression ensemble combined Random Forest (4.7-1.2), Gradient Boosting, and XGBoost (3.1.0) to predict tensile strength from thermal patterns. Random Forest captured nonlinear relationships, Gradient Boosting corrected systematic biases, and XGBoost added regularization. The ensemble averaged predictions from all three models equally. An ensemble was used to reduce overfitting risk and improve prediction stability in the small-sample regime by combining complementary tree-based regressors (bagging and boosting).

We selected features based on moderate (not dominant) importance scores to improve generalization beyond the training dataset. Through prediction importance analysis, we targeted features representing temporal evolution (thermal buildup consistency across layers), radial decay patterns (physical diffusion characteristics), and cross-layer consistency (process stability). We intentionally excluded features with very high importance that likely represented experimental setup inconsistencies, such as absolute thermal magnitudes from outside factors, specific spatial locations, or printer-specific patterns. This ensured that we eliminated unimportant features to focus on fundamental thermal–mechanical relationships.

We standardized features (zero mean, unit variance) before training to equalize the influence of features with different units and ranges. The individual models (that would later be combined for one ensemble model) used conservative hyperparameters:

*Random Forest:* 200 trees, maximum depth 10;

*Gradient Boosting:* Maximum depth 5, learning rate 0.1;

*XGBoost:* L1 and L2 regularization (α = 0.1, λ = 0.1), Early stopping to prevent overtraining.

We used the same 80/20 stratified split as the classification model for consistency.

Five-fold cross-validation with stratified partitioning assessed performance comprehensively, with each fold training on 24 specimens and validating on 6. We evaluated models using R^2^ (explained variance), MAE (average prediction error), and RMSE (sensitivity to outliers), with confidence intervals calculated across folds to assess stability.

## 3. Results

*Mechanical Testing:* mechanical testing of the 30 specimens revealed relatively similar strengths within the varying print conditions (Table 3).

Standard deviations in the data were relatively small, with the lowest value being 0.13 MPa for B (Standard conditions) and the highest value being 0.76 MPa for D (the Asymmetric condition). The higher variability observed in Condition D is expected because the asymmetric heating/cooling configuration generates an inherently unstable convective environment. Opposing hot and cold air streams can form recirculation zones and intermittent vortices around the print, producing time-dependent and spatially non-uniform cooling rates. As a result, small differences in specimen position, airflow alignment, or printing time history can lead to appreciable variation in local thermal history within the gauge section, affecting interlayer diffusion and residual stress development. This condition therefore promotes greater specimen-to-specimen variability than symmetric cooling/heating cases, which provide more uniform and repeatable thermal boundary conditions.

Although six distinct environmental conditions were imposed, the measured ultimate tensile strengths remained relatively close across groups. This outcome is expected because the thermal history that governs PLA interlayer diffusion is dominated by the nozzle temperature and local melt deposition, while the external airflow conditions primarily influence the surrounding convective field and cooling boundary layer. As a result, the imposed heating/cooling conditions may have been sufficient to generate distinct Schlieren signatures without producing large, statistically separable changes in bulk tensile strength for this geometry and material. Furthermore, the modest sample size (*n* = 5 per condition) limits statistical power for detecting small mechanical shifts.

From a statistical standpoint, the narrow spread in tensile strength values suggests that the imposed environmental conditions did not produce large shifts in ultimate tensile strength under the present sample size (*n* = 5 per condition). With such modest group sizes, only relatively large effect sizes would be expected to reach conventional statistical significance (e.g., α = 0.05). Therefore, while condition D exhibited the largest variability and some differences in mean values are observed, the dataset primarily supports the conclusion that the environmental modifications created measurable thermal field differences without causing dramatic tensile strength separation for this PLA geometry and printing configuration. Importantly, this outcome does not imply that the thermal environment is irrelevant; rather, it indicates that the mechanical response is influenced by multiple competing factors and that the effect magnitude may be small compared to the inherent strength level of the prints.

**Figure 4:**
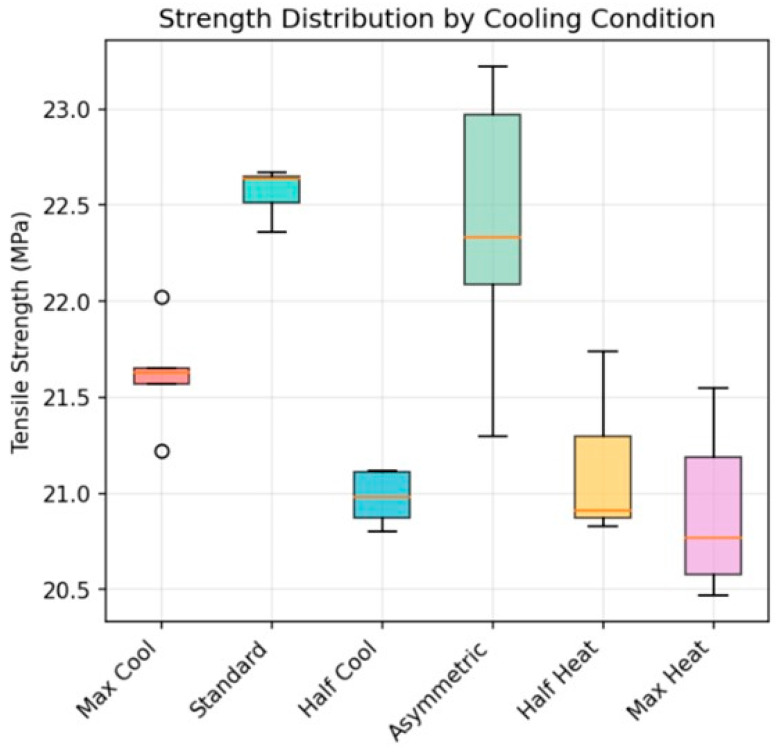
Tensile strength of test specimen (orange lines show the average values).

Figure 4 is a visualization of this data using different statistical terms. Each box represents the interquartile range (25th–75th percentiles) with the median indicated by the horizontal line within each box. Outliers are shown as individual points. The median provides a robust indicator of the typical tensile strength for each condition and allows comparison of central tendency without being overly influenced by any single specimen. The interquartile range (IQR) quantifies the spread of the middle 50% of measurements and therefore reflects the repeatability and consistency of tensile strength under each printing environment. Conditions with a narrower IQR indicate more uniform mechanical performance (higher process stability), whereas a wider IQR indicates increased variability in print-to-print strength, consistent with greater thermal and airflow fluctuations during fabrication. In Figure 4, the relatively small IQRs across most conditions suggest comparable mechanical consistency, while the larger spread in Condition D supports the conclusion that asymmetric thermal boundary conditions introduce higher variability.

Schlieren Imaging Outputs: The BOS processing algorithm transforms paired reference and test images into comprehensive visualizations of analytical outputs. The program generates three primary visualizations: density gradients that provide heat maps of optical disturbances, pixel magnitude shift distribution graphs that quantify the statistical characteristics of the displacement field, and detailed CSV files containing pixel-level displacement vectors specifying both magnitude and directional components for every pixel coordinate in the analyzed region.

Density gradient visualizations served as a quality control tool for preliminary data screening, allowing for the removal of outliers in the dataset. These visualizations employ adaptive color scaling, where the color map dynamically adjusts to the magnitude range within each image.

**Figure 5:**
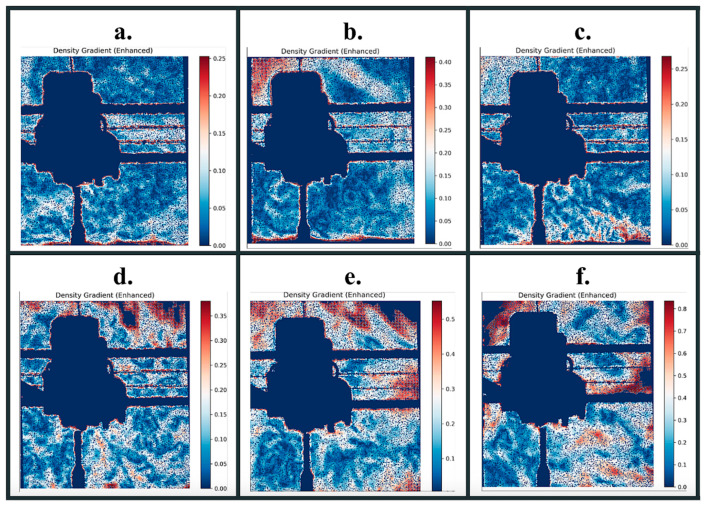
Schlieren image outputs: (**a**) maximum cooling, (**b**) standard environment, (**c**) external half cooling, (**d**) asymmetric environment, (**e**) external half heating, and (**f**) maximum heating.

Consequently, identical colors represent different absolute values across conditions; for instance, red regions in a “Max Cool” image correspond to different density gradient magnitudes than red regions in a “Max Heat” image. Figure 5 presents representative Schlieren density gradient visualizations across all environmental conditions at layer 275, demonstrating the range of thermal and flow phenomena captured under different experimental parameters.

**Figure 6:**
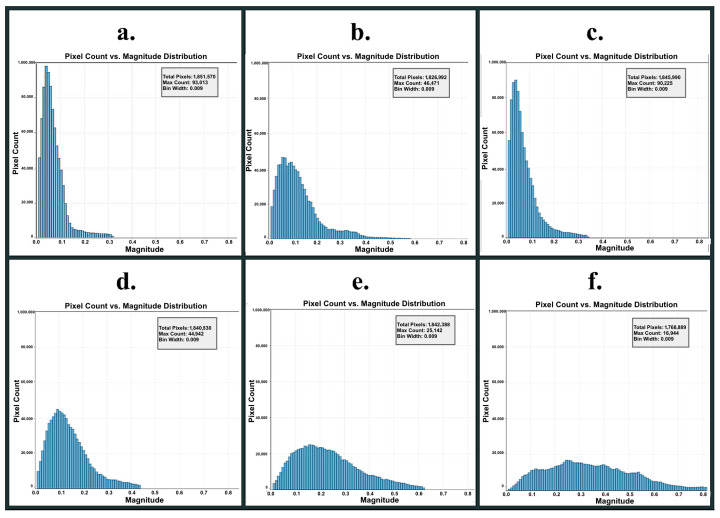
Pixel magnitude shift distribution (PSMD) histograms extracted from BOS–Schlieren optical flow displacement fields for the six environmental printing conditions (**a**–**f**). Each plot shows pixel count versus displacement magnitude (bin width = 0.009), where higher magnitudes indicate stronger convective thermal gradients around the print: (**a**) maximum cooling, (**b**) standard environment, (**c**) external half cooling, (**d**) asymmetric environment, (**e**) external half heating, and (**f**) maximum heating.

Pixel Shift Magnitude Distribution (PSMD) graphs provide a strong visualization for analyzing the Schlieren image data (Figure 6). These distributions exhibit a striking resemblance to Maxwell–Boltzmann distributions, revealing an underlying physical analogy between molecular kinetic behavior and optical flow patterns. This data was later used in the ensemble machine learning models to classify the conditions under which specimens were printed and ultimately predict tensile strength.

Machine Learning Model Performance (80/20 Train/Test Split): The successful classification of printing conditions served as a crucial validation metric for model performance in this study. Each condition (max cool, standard, half cool, asymmetric, half heat, and max heat) generated characteristic Schlieren signatures and unique pixel magnitude shift distribution profiles, demonstrating the potential of the optical measurement technique.

**Figure 7:**
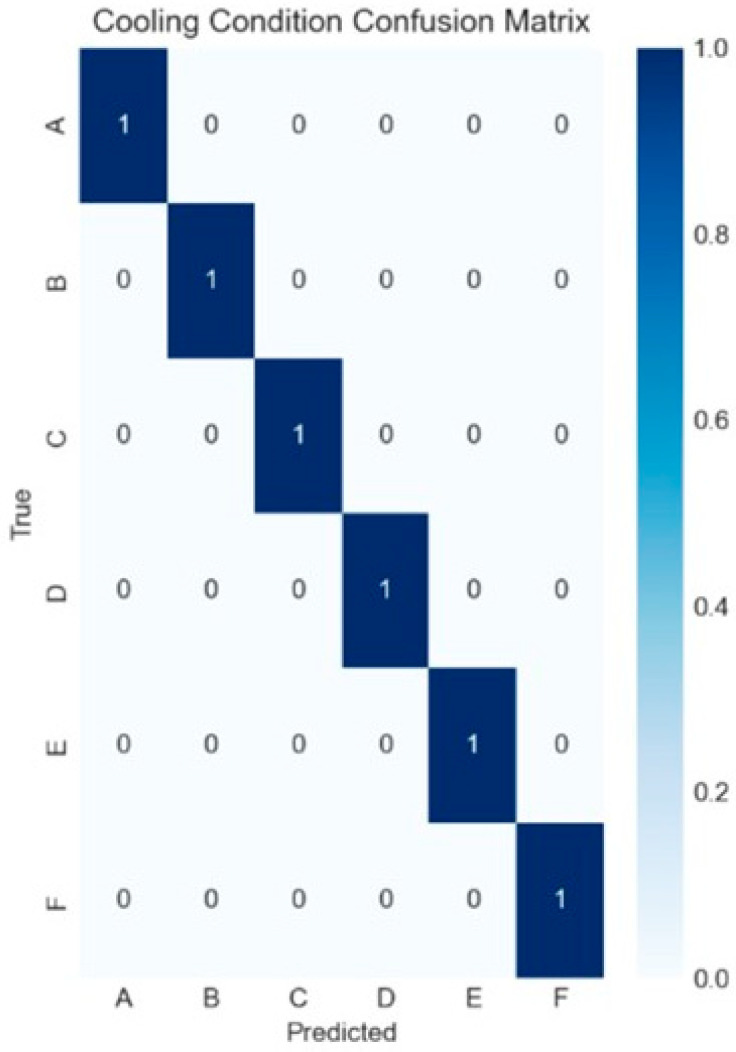
Confusion matrix for the cooling condition classification model (conditions A–F), showing perfect agreement between true and predicted labels (normalized diagonal values = 1.0; no misclassifications).

To establish the reliability of the Schlieren-derived features, an ensemble classification approach was used prior to tensile strength prediction. The ensemble combined Random Forest and XGBoost classifiers to identify the printing conditions from Schlieren data inputs. Using nine Schlieren-derived CSV feature sets and an 80/20 train–test data split, the classification model achieved perfect accuracy (100%) in condition identification (Figure 7). While the 80/20 split yielded 100% classification accuracy, such performance can be sensitive to dataset size and split selection and may reflect overfitting risk in small-sample studies. Therefore, model generalization was evaluated using five-fold stratified cross-validation, which achieved 90% accuracy, providing a more conservative estimate of performance on unseen data.

Tensile Strength Prediction: Beyond classifying the environmental condition, the model proved successful in predicting tensile strength with an 80/20 train-test split (meaning the model was trained on 24 specimens and tested with six specimens).

**Figure 8:**
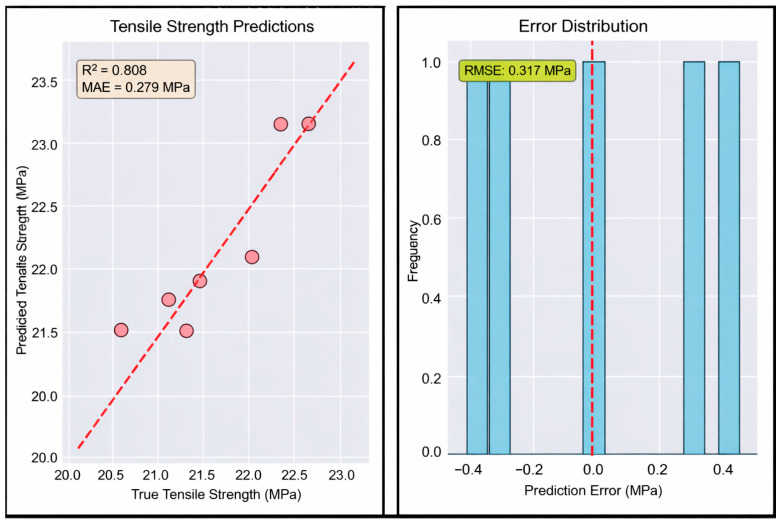
Tensile strength prediction performance for the 80/20 train–test split regression model using Schlieren-derived thermal fingerprint features. (**Left**) Predicted tensile strength versus true tensile strength, with the dashed line indicating the ideal 1:1 agreement; the model achieved R^2^ = 0.808 and MAE = 0.279 MPa. (**Right**) Distribution of prediction errors (predicted − true), showing errors centered near zero with RMSE = 0.317 MPa (dashed line), indicating good predictive accuracy on the held-out test set.

An ensemble regression approach was implemented; the ensemble combined a Random Forest Regression with 200 trees, Gradient Boosting Regressor with 200 estimators, and XGBRegressor with 200 estimators to estimate the final tensile strength from Schlieren data inputs. Using Schlieren-derived CSV features at nine layers for each sample and an 80/20 train–test data split, the model achieved accuracy with a mean absolute error (MAE) of 0.279 MPa and an R^2^ value of 0.808 as shown in Figure 8.

To assess model generalizability, k-fold cross-validation (KFCV) with five folds was implemented as a robust evaluation framework. This approach splits up the dataset into five equal subsets, iteratively training on four folds while validating on the remaining fold. This provides a more generalizable estimate of model performance on unseen data.

**Figure 9:**
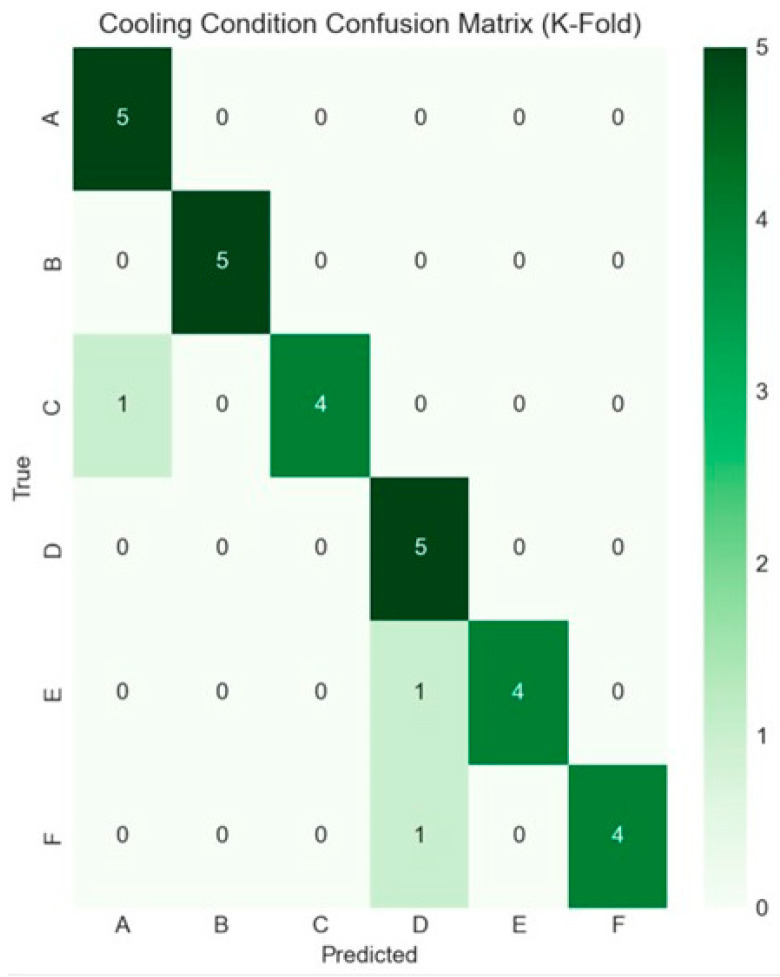
Confusion matrix for five-fold cross-validation cooling condition classification (conditions A–F) using Schlieren-derived BOS thermal fingerprint features. Values represent the total number of predictions aggregated across folds. The strong diagonal dominance indicates robust generalization across folds, with an overall cross-validated accuracy of 90%; limited misclassifications occur primarily between conditions with more similar thermal field signatures.

**Figure 10:**
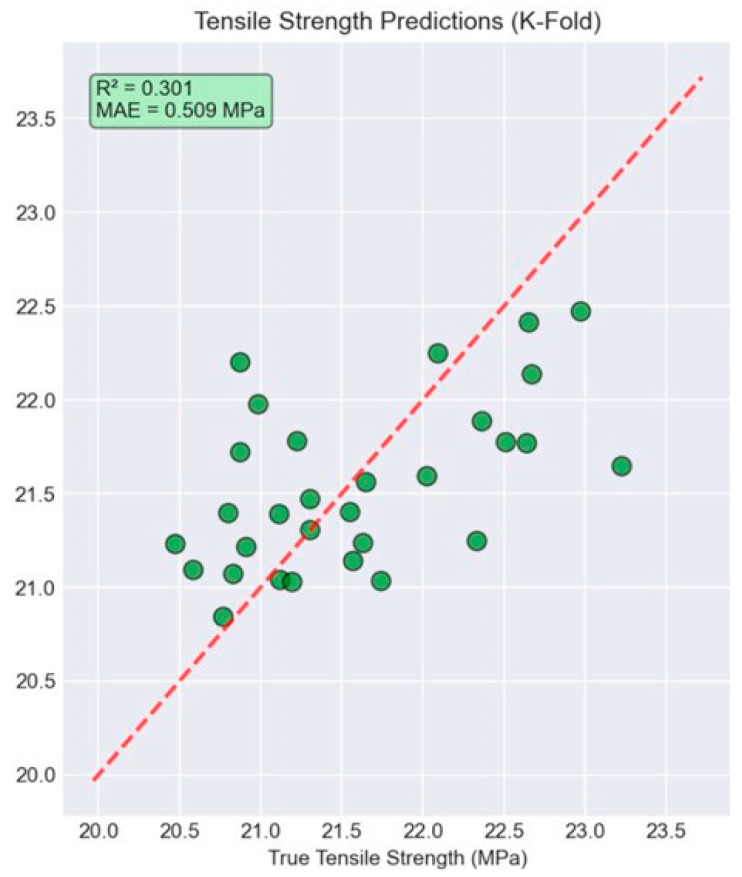
Tensile strength prediction results using five-fold cross-validation with Schlieren-derived thermal fingerprint features. Predicted tensile strength is plotted against the experimentally measured values for all specimens across folds, with the dashed line representing ideal 1:1 agreement. Cross-validation yields R^2^ = 0.301 and MAE = 0.509 MPa, indicating that while the BOS–Schlieren features contain predictive signal, regression generalization is more challenging under limited sample size and fold-to-fold variability.

The cross-validation results revealed nuanced performance characteristics across the two prediction tasks. For condition classification, the model maintained strong generalization capability with 90% accuracy (Figure 9), representing only a 10% reduction from the initial perfect training performance. However, the tensile strength prediction task proved more challenging under cross-validation conditions, achieving an MAE of 0.509 MPa (Figure 10) and an R^2^ value of 0.301.

The regression performance under cross-validation (MAE = 0.509 MPa, R^2^ = 0.301) reflects the constraints of a small dataset (*n* = 30), where each fold trains on only 24 samples, increasing sensitivity to data partitioning and limiting generalization. Nevertheless, the observed MAE corresponds to only a few percent of the average tensile strength, indicating that Schlieren-derived thermal fingerprints contain meaningful predictive signals. Improving robustness will require larger and more diverse datasets, stronger dimensionality reduction and regularization to reduce model variance, and validation strategies that better represent real-world deployment (e.g., repeated stratified CV and leave-one-printer/session-out testing).

## 4. Discussion

Interpretation of Findings: The results of this study demonstrate that thermal gradients captured through Background-Oriented Schlieren imaging contain information about the mechanical properties of FDM-printed PLA specimens. The successful prediction of tensile strength (MAE = 0.279 MPa, R^2^ = 0.808) in the 80/20 train–test split validates the core hypothesis that thermal fingerprints have meaningful data about interlayer bonding quality and resulting ultimate tensile strength performance.

This relationship can be understood through the fundamental physics of FDM. The tensile strength of FDM parts is primarily determined by interlayer adhesion, which depends on the thermal environment present during printing. When a new layer is deposited, polymer chains must achieve sufficient molecular mobility to diffuse across the previous layer and form entanglements with it. Thermal gradients play a central role in this process because they directly control the local cooling rate and the duration that the deposited filament remains above critical transition temperatures (e.g., glass transition, Tg). When cooling is slow and the interfacial temperature remains elevated for longer, polymer chains retain higher molecular mobility, enabling greater reptation and interdiffusion across the layer interface. This increases entanglement density between adjacent rasters/layers, forming a stronger weld line and improving ultimate tensile performance. Conversely, steeper thermal gradients and rapid cooling reduce the time available for interfacial diffusion, causing the melt to solidify sooner and “freeze in” a weaker interlayer bond with reduced chain entanglement. In addition, nonuniform thermal gradients can generate residual stresses and localized shrinkage mismatch, which further degrade interlayer adhesion and increase the likelihood of premature crack initiation along interfacial regions. Therefore, the convective thermal environment captured through BOS imaging provides physically meaningful information about molecular mobility history and resulting interlayer bond quality in FDM prints. While the mean strengths across conditions were similar, the thermal environments can still influence tensile behavior by altering cooling rate, interlayer diffusion depth, residual stress development, and local bead geometry/void formation. These factors may lead to subtle differences in bonding quality that do not necessarily manifest as large changes in mean tensile strength for small datasets, but they do produce measurable differences in the surrounding air-side thermal gradients. This helps explain why BOS-derived features reliably classified environmental conditions and still contained predictive signals for tensile strength.

The model’s reliance on features such as near-field thermal variability and radial zone characteristics indicates that both the magnitude and spatial distribution of thermal gradients contain information for predictions. Radial features describe how thermal energy dissipates from the print location, and different decay patterns correspond to different environmental conditions that ultimately affect cooling rates and bond formation.

High classification accuracy (100% in 80/20 split, 90% in cross-validation) demonstrates that varying environmental conditions generate distinct thermal signatures. However, regression performance under cross-validation (R^2^ = 0.301, MAE = 0.509 MPa) reveals traditional limitations in predicting mechanical properties from small datasets. The small dataset (*n* = 30) and high feature count create overfitting risk despite regularization. Still, reasonable performance was maintained (2.3% error relative to mean strength). This suggests that a predictive signal exists, but larger datasets would be required for robust generalization.

Comparison with Prior Work: Prior in situ thermal monitoring efforts in material extrusion/FFF have primarily focused on surface temperature measurements (most commonly infrared thermography) and/or parameter-driven surrogates of thermal history. While these approaches are valuable for detecting process anomalies, they face recurring limitations when used to predict mechanical properties. First, surface temperature does not directly capture the air-side convective boundary layer and surrounding flow structures that govern cooling rate and interlayer thermal history, which can lead to incomplete representation of the bonding conditions [7]. Second, many thermal-history studies rely on process-specific calibration (camera angle, emissivity assumptions, enclosure configuration, or printer-dependent heat accumulation), which can limit transferability to new geometries, machines, and ambient conditions. Third, even when thermal features correlate with quality outcomes, predicting mechanical properties remains challenging because strength is influenced by multiple coupled mechanisms (interlayer diffusion/entanglement, void formation, residual stresses), and small datasets often yield optimistic holdout performance that degrades under cross-validation or external testing. Finally, several frameworks predict intermediate metrics (e.g., consolidation degree or thermal indices) rather than directly validating against mechanical testing across diverse conditions, leaving a gap in robust, part-specific property prediction. 

In contrast, the present study targets air-side thermal gradients via BOS/Schlieren-derived fingerprints, which are physically linked to convective cooling behavior and provide complementary information to surface thermography for property prediction. Schlieren imaging advances beyond infrared thermography by capturing volumetric thermal gradients in surrounding air rather than just surface temperatures. This distinction is critical because cooling rates depend on thermal boundary layers and convective heat transfer, not surface temperature alone. The asymmetric heating condition especially proves this: competing airflows create complex gradient patterns reflecting the actual convective environment. This is information that surface measurements cannot provide. Additionally, BOS implementation costs under USD 1000 compared to USD 10,000–USD 50,000+ for high-speed IR systems, making thermal monitoring more accessible to smaller manufacturers.

Printing-parameter-based models predict part properties from process settings without additional hardware, seeming to offer practical baseline predictions. However, these models assume that identical parameters produce identical parts. This assumption is violated by environmental variations and equipment differences. Thermal fingerprinting directly measures the actual thermal environment experienced by each part. It captures variations that parameter tables cannot represent. This suggests hybrid approaches where parameter-based baselines are refined using these volumetric thermal measurements for part-specific accuracy.

To the authors’ knowledge, this represents the first application of Schlieren imaging to quality assessment in polymer additive manufacturing. While Schlieren techniques are established in aerodynamics and heat transfer research, and explored for metal additive manufacturing, the polymer additive manufacturing community has not previously used this technique for tensile strength prediction. This establishes a proof-of-concept for an entirely new class of non-destructive testing in FDM.

Limitations: The study’s primary limitation is the small dataset (*n* = 30, with *n* = 5 per condition). It limits statistical power and creates a high feature-to-sample ratio, which contributes to overfitting risk and cross-validation performance reduction. Generalization is limited by material specificity (single PLA grade), simple geometry (dogbone specimens), fixed process parameters (200 °C, 60 mm/s, 20% infill), and somewhat artificial environmental conditions. Real production environments exhibit subtler variations that may prove more challenging to detect. Further statistical analysis could include one-way ANOVA (or Kruskal–Wallis if normality is not assumed) to test between-condition differences, along with effect size metrics (η^2^ or Cohen’s d) and post hoc comparisons (Tukey HSD). In addition, correlating tensile strength directly with BOS-derived metrics (e.g., thermal variability and asymmetry indices) would provide a quantitative bridge between thermal fingerprints and mechanical outcomes beyond group-based comparisons.

Mechanical testing focused exclusively on ultimate tensile strength in one loading direction, while real-life applications require diverse properties including modulus, toughness, and fatigue resistance under multi-directional loading. Also, the single-camera configuration cannot fully resolve three-dimensional thermal fields, and the nine-layer sampling captures temporal evolution but remains limited relative to 400-layer prints. Finally, while demonstrating correlation, the study does not fully establish the link between specific thermal patterns and mechanical outcomes. The machine learning models function as black boxes without explicit physics. In practical manufacturing contexts, even small deviations in strength (on the order of 1–3%) can be meaningful for parts that operate near design allowables or under fatigue loading. Thus, the relatively similar mean strengths observed here do not reduce real-world relevance; instead, they demonstrate that thermal fingerprints can capture part-specific process variation even when mechanical changes are subtle. This is precisely the regime where non-destructive, in situ quality monitoring is valuable—identifying prints that deviate from expected behavior before destructive testing or failure occurs.

While the classification model demonstrated strong performance (90% accuracy under five-fold cross-validation), improving generalization for real-world deployment will require broader datasets and validation under domain shift. Future work will expand the dataset across additional printer platforms, print sessions, ambient conditions (temperature, humidity, enclosure state), filament batches, and part geometries to reduce sensitivity to setup-specific features. In addition to k-fold cross-validation, more deployment-realistic validation schemes will be explored, including leave-one-condition-out, leave-one-print-session/day-out, and leave-one-printer-out testing to directly assess robustness to unseen environments and hardware. Generalization will also be improved by emphasizing physically meaningful normalized descriptors (e.g., radial profiles, asymmetry indices, percentile-based statistics) and reducing dependence on absolute magnitude or fixed spatial-coordinate features that may encode nuisance factors such as lighting or camera placement. Finally, uncertainty-aware classification (e.g., probability calibration and out-of-distribution detection) will be incorporated to flag “unknown” operating regimes and prevent overconfident misclassification in production settings.

## 5. Conclusions

This study introduced AI-powered thermal fingerprinting as a proof-of-concept framework for predicting the mechanical performance of FDM-printed polymers using Background-Oriented Schlieren (BOS) imaging and machine learning. By capturing air-side thermal gradients during printing, this method provides a low-cost, non-contact pathway for in situ monitoring of convective thermal environments that influence print quality.

Key outcomes from this work include the following:

1. Successful thermal field capture using low-cost BOS instrumentation:

The BOS setup reliably produced Schlieren displacement fields across 30 PLA specimens printed under six controlled environmental conditions, generating a dataset of 270 high-resolution thermal field images (nine layers per specimen).

2. Clear thermal separability of printing environments:

Schlieren-derived features enabled highly accurate identification of environmental conditions. The ensemble classification model achieved 100% accuracy in an 80/20 split and 90% accuracy under five-fold stratified cross-validation, demonstrating that BOS-based thermal fingerprints contain strong condition-specific signatures.

3. Feasibility of tensile strength prediction from thermal fingerprints:

The ensemble regression model demonstrated promising tensile strength prediction performance in the 80/20 split (R^2^ = 0.808, MAE = 0.279 MPa). More rigorous evaluation using five-fold cross-validation yielded R^2^ = 0.301 and MAE = 0.509 MPa, highlighting both the presence of predictive signals and the limitations imposed by small datasets for robust regression generalization.

Overall, these findings validate the central premise of this work: thermal gradients surrounding the print encode measurable information relevant to interlayer bonding and tensile strength, and BOS-based Schlieren imaging can serve as a practical sensing method for extracting that information. While the study is constrained by dataset size, single material (PLA), and simplified geometry, it establishes a reproducible methodology for linking air-side convective thermal behavior with mechanical property outcomes.

Future work will scale the dataset across additional polymers, geometries, and printer platforms, and will incorporate deployment-oriented validation (e.g., leave-one-printer/session-out testing) to strengthen generalization. With these advancements, thermal fingerprinting has the potential to support real-time, non-contact quality assurance for polymer additive manufacturing.

Despite these promising results, this work remains a proof-of-concept study with several limitations. The dataset size was limited (*n* = 30 specimens, *n* = 5 per condition), which restricts statistical power and contributes to reduced regression generalization under cross-validation. The study also focused on a single material (PLA), a single specimen geometry, and fixed printing parameters, while the imposed environmental conditions represent simplified thermal scenarios compared to real production environments. Additionally, BOS imaging was performed at nine selected layers rather than continuously across the full print, and the single-camera configuration does not resolve fully three-dimensional thermal fields. Future work will address these limitations by expanding datasets to hundreds or thousands of specimens across multiple polymers (e.g., PETG, ABS, TPU), printer platforms, filament batches, ambient conditions, and more complex part geometries. More deployment-relevant validation strategies (e.g., repeated stratified cross-validation and leave-one-printer/session-out testing) will also be implemented to quantify robustness under domain shift. Finally, integrating physics-informed feature engineering and uncertainty-aware modeling is expected to improve predictive reliability and support scalable, real-time quality assurance for polymer additive manufacturing.

## Figures and Tables

**Table 1:** Definition of environmental conditions for the samples that were 3D printed.

Condition	Name	Description
A	Maximum Cooling	Two hairdryers positioned on opposite sides of the printer operated on “maximum cooling” settings. This created the lowest ambient temperature environment.
B	Standard Environment (Control)	Only the printer’s internal fan provided airflow. This represented typical consumer printing conditions with minimal external alterations.
C	Moderate Cooling	Two hairdryers operated on “reduced cooling” settings. This provided moderate cooling between maximum cooling and standard conditions.
D	Asymmetric Thermal Environment	One hairdryer operated on maximum heating (right side) while the other one operated on maximum cooling (left side). This generated complex airflow patterns to simulate complex vortexes within environments.
E	Moderate Heating	Two hairdryers operated on “reduced heating” settings. This provided moderately heated thermal environments.
F	Maximum Heating	Two hairdryers operated on “maximum heating” settings. This created the highest temperature environment.

**Table 2:** Random Forest and XGBoost hyperparameters for the ML study.

Random Forest Hyperparameters			
**Number of Trees**	**Maximum Depth**	**Minimum # of Samples per Leaf**	
200	10	1	
**XGBoost Hyperparameters**			
**Number of Trees**	**Maximum Depth**	**Minimum # of Samples per Leaf**	**L2 Regularization**
200	6	1	α = 0.01

**Table 3:** Tensile testing results for FDM-printed PLA specimens under six environmental printing conditions (A–F).

Tensile Strength (MPa)						
	A	B	C	D	E	F
1	21.22	22.51	20.80	21.30	20.83	20.47
2	21.65	22.67	21.12	23.22	20.91	20.58
3	21.57	22.36	21.11	22.09	20.87	21.19
4	22.02	22.64	20.98	22.97	21.74	20.77
5	21.63	22.65	20.87	22.33	21.30	21.55
Mean:	21.62	22.57	20.98	22.38	21.13	20.91
Standard Deviation:	0.28	0.13	0.14	0.76	0.39	0.45
Standard Deviation Percent of Average:	1.32	0.58	0.68	3.39	1.84	2.1

## Data Availability

The original contributions presented in this study are included in the article. Further inquiries can be directed to the corresponding author.
